# Prenatal ultrasound manifestations of partial urorectal septum malformation sequence from the first trimester to postnatal: a case report

**DOI:** 10.1186/s12884-023-05939-1

**Published:** 2023-08-24

**Authors:** Lingna She, Hualan Lin, Shuxian Huang, Lina Liu, Liyan Chen

**Affiliations:** 1 Department of Ultrasound, Meizhou People’s Hospital, NO. 63 Huangtang Road, Meizhou, China; 2 Department of Obstetrics, Meizhou People’s Hospital, Meizhou, China

**Keywords:** Ultrasound, Urorectal septum malformation sequence, Duplicated vagina and uterus, Anal atresia, Hydronephrosis, Ascites

## Abstract

**Background:**

Urorectal septum malformation sequence (URSMS) is characterized by incomplete partitioning of the genital, rectal, and urinary tracts, resulting in a severe form of anorectal malformation. The partial URSMS, also known as the persistent cloaca, represents a milder variant where a single perineal opening serves as a passage for the urinary, gastrointestinal, and reproductive tracts.

**Case presentation:**

We present a rare case of partial URSMS accompanied by duplicated vagina and uterus, hydronephrosis, ascites, and anal atresia.

**Conclusions:**

This case report describes the sonographic findings at different stages of pregnancy and their changes throughout gestation.

## Background

Urorectal Septum Malformation Sequence (URSMS) is a series of congenital abnormalities resulting from a migration/fusion disorder of the urorectal septum and maldevelopment of the cloacal membrane. It is characterized by the absence of perineal and anal openings, ambiguous vulvar sex, abnormalities in urinary and genital structures, colon malformations, lumbosacral, and other abnormalities [1]. It is a rare anomaly with a prevalence of 1:50 000 to 1:250000 live births [2].URSMS can be categorized into complete and partial forms based on the presence or absence of cloaca membrane breakdown [1]. Partial URSMS refers to direct communication between the gastrointestinal, urinary, and genital structures, forming a single perineal opening known as the persistent cloaca, predominantly observed in females. We report a case of a patient diagnosed with partial URSMS who underwent prenatal ultrasound examinations starting at 12 weeks of gestation. However, the diagnosis was not made until birth.

In our case, positive findings were observed on ultrasonography from 12 weeks of gestation, and these findings progressively worsened as gestational weeks advanced. Before delivery, the case was diagnosed as “anal atresia,“ “meconium peritonitis,“ and “hydronephrosis.“ However, after birth, it was confirmed as a case of partial URSMS. This paper aims to analyze the ultrasound manifestations and characteristics of the patient throughout pregnancy to enhance understanding of partial URSMS and improve the rate of prenatal diagnosis.

## Case presentation

A 38-year-old pregnant patient, gravida 2, para 1, was directed to our Prenatal Diagnosis Center at 12 weeks of gestation due to suspicion of an abdominal cystic mass in the fetus. Apart from one previous normal vaginal delivery, the patient’s obstetric history was unexceptional. The diagnosis of gestational diabetes was made following a glucose tolerance test conducted around the 24th week of gestation. After diabetes management during pregnancy, her blood sugar was well controlled. Moreover, there was no reported family history of congenital anomalies. The mother reported no exposure to teratogenic substances. The Serologic test for TORCH (Toxoplasmosis, Rubella, Cytomegalovirus, Herpes simplex virus) infections revealed a negative result.

**Fig. 1:**
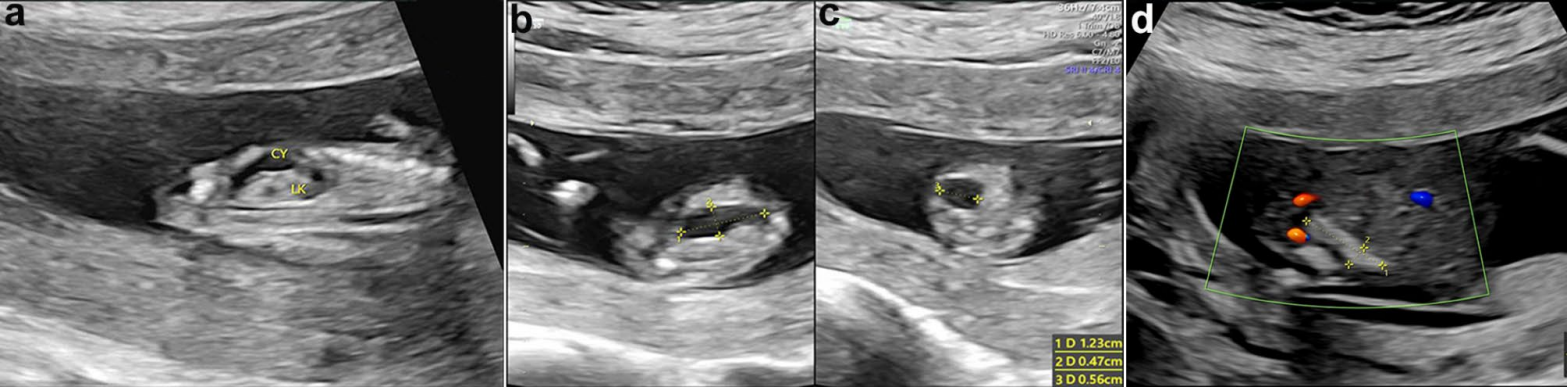
Ultrasound (US) images show the evolution of fetal abdominal cyst (CY) from 12 and 15 weeks of gestation, respectively. (**a**) The sagittal US image of the fetal abdomen depicts a bowel-like cystic structure lying anterior to the left kidney (LK) at 12 weeks of gestation. Transverse (**b**) and longitudinal (**c**) views of the cystic structures measuring 12 × 6 × 6 mm (cursors). (**d**) Follow-up ultrasound examination at 15 weeks of gestation shows the echogenic bowel in the left lower abdomen measuring 16 × 4 mm (cursors); the cyst was not visible

Grayscale fetal ultrasonography (US) revealed an anechoic, unilocular, sausage-shaped mass in the mid-abdomen, lying anterior to the left kidney (Fig. 1a), measuring 12 × 6 × 6 mm (Fig. 1b, c). The nuchal translucency measured 1.3 mm. No significant abnormalities were found in the urinary bladder or stomach, and no other notable fetal abnormalities were detected. A repeated fetal ultrasound was performed at 15 weeks of gestation. It showed that the cyst was not visible, echogenic bowel in the left lower abdomen with an area of 16 × 4 mm (Fig. 1d). Based on the abnormal ultrasound findings, an amniocentesis procedure was conducted, which confirmed a normal karyotype with G-banding analysis, showing a 46 XN pattern. The prenatal chromosome microarray analysis revealed a chromosomal composition of arr (1–22) × 2, (XN) × 1.

**Fig. 2:**
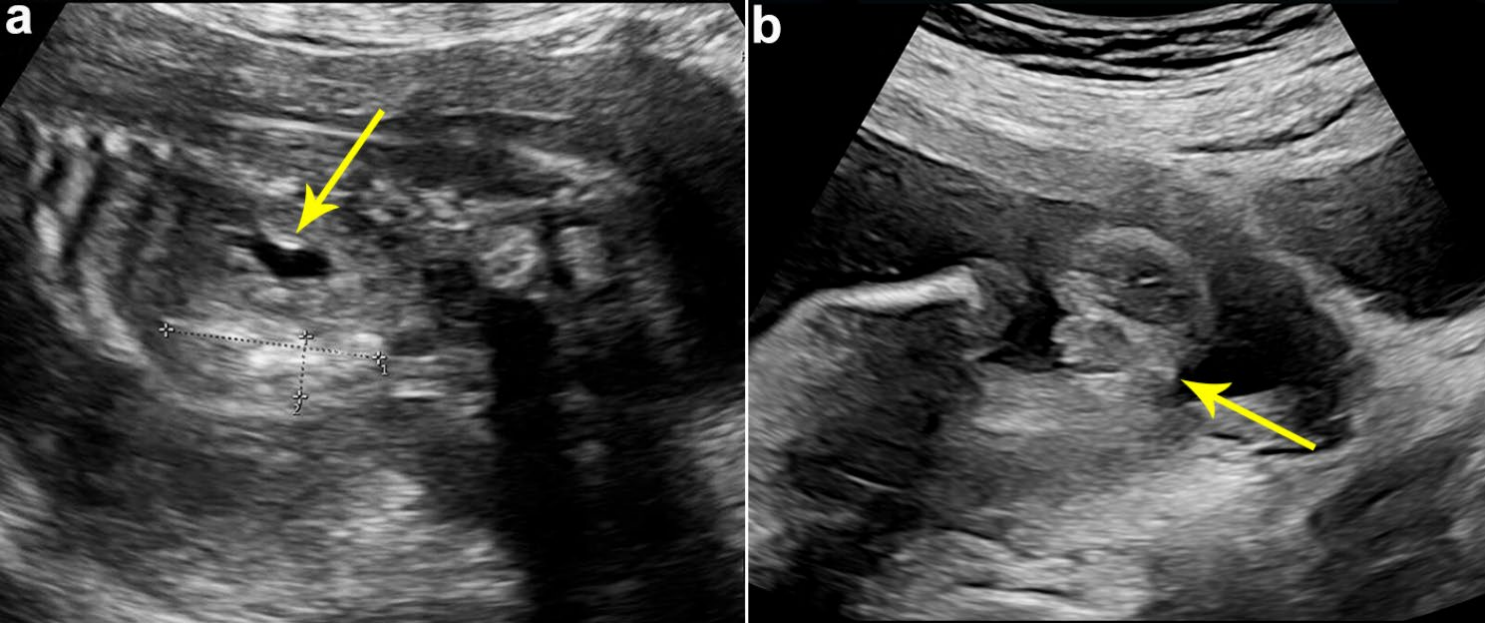
Ultrasound image at 23 weeks of gestation. (**a**) The echogenic bowel (cursors) and mild hydronephrosis of the left kidney (arrow). (**b**) The transverse sonogram of the perineum indicates the absence of a hypoechoic ring (arrow), suggesting the presence of an invisible anal sphincter

**Fig. 3:**
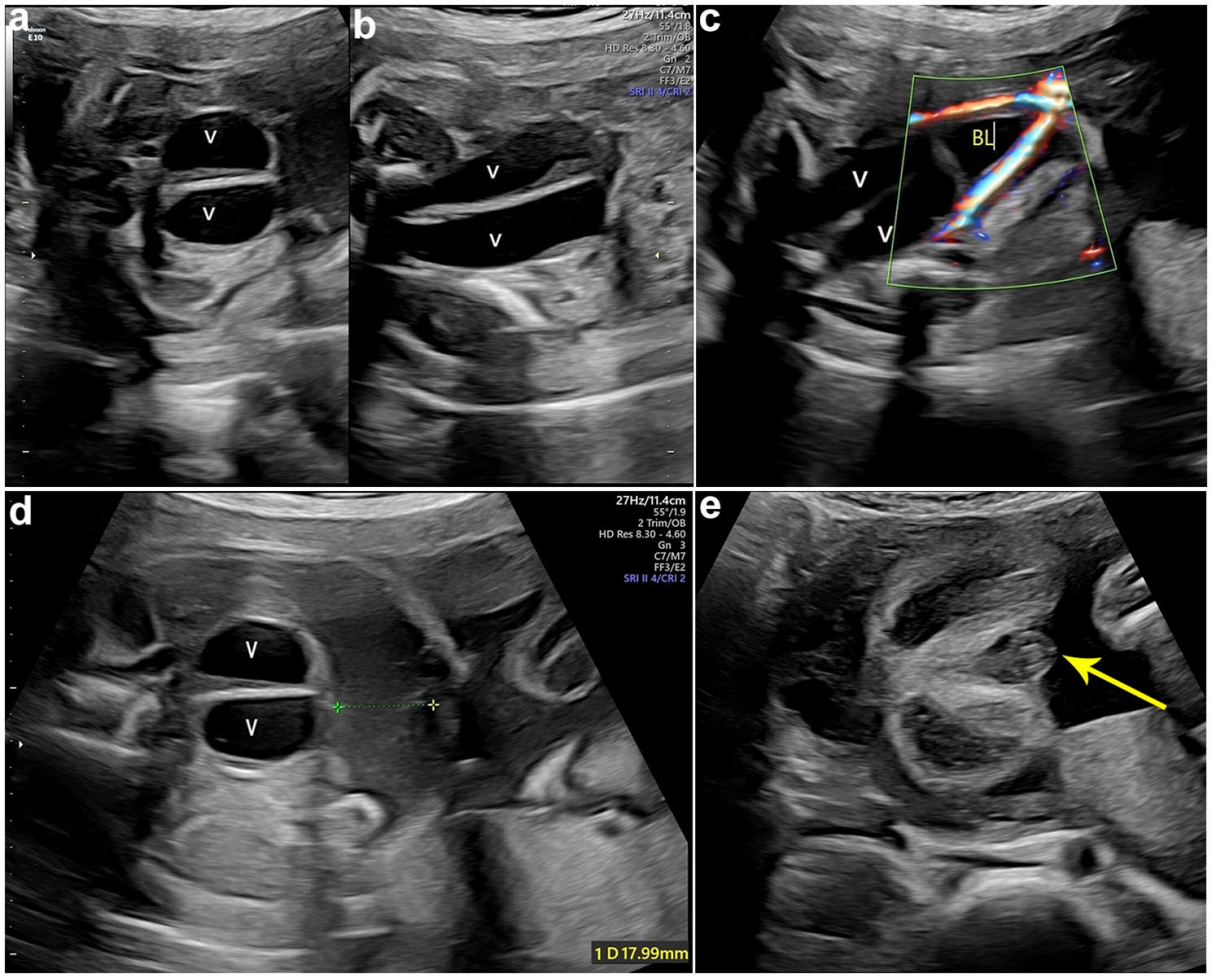
Ultrasound image at 27 weeks of gestation. Transverse (**a**) and longitudinal (**b**) views of the fetal pelvis show a centrally septated tubular structure, indicating duplicated and fluid-filled vaginal canals (V). (**c**) Centrally septated tubular structure behind the bladder (BL) that represents duplicated dilated vaginal canals(V). Doppler is used to delineate bladder position. (**d**) Transverse views of the fetal pelvis, showing duplicated vaginal canals (V) and fetal ascites (cursors). (**e**) Transverse sonogram of perineum hypertrophy of clitoris (arrow)

**Fig. 4:**
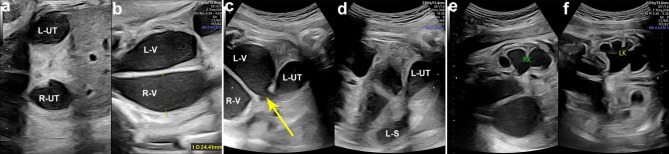
Axial images of the abdomen and pelvis at 31 and 33 weeks of gestation. Hydrometrocolpos of the duplicated uterus (**a**) and vaginal canals (**b**) at 31 weeks of gestation. The duplicated uterus is located on the left and right sides of the pelvic cavity and arranged symmetrically (**a**). (**c**) Left vaginal connected to left uterus, the junction is the cervix(arrow), and (**d**) the left uterus connected to left hydrosalpinx at 33 weeks of gestation. (**e**, **f**) Bilateral moderate to severe hydronephrosis with mild renal parenchymal thinning. LUT, left-sided bicornate uterus; LS, left hydrosalpinx; R-UT, right-sided bicornate uterus; RK, right-sided kidney; LK, left-sided kidney

**Fig. 5:**
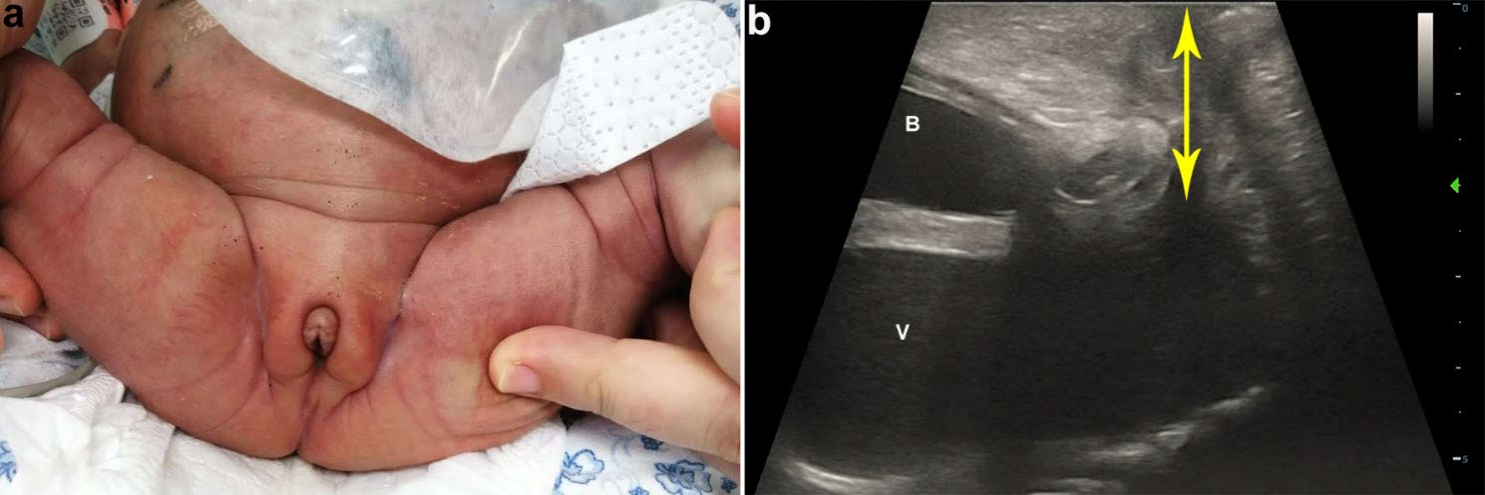
Photo and transperineal ultrasound images of the perineum of newborns. (**a**) Newborn image shows a single perineal opening where the urethra is expected and the absence of an anal orifice. (**b**) Sagittal sonogram obtained using a perineal approach on the 34th day after birth. A common canal (double-headed arrow) with a length of about 30 mm. B, bladder; V, vaginal

At 23 + weeks of gestation, the fetal ultrasound displayed that the echogenic bowel was still present and mild dissociation of the left renal pelvis (Fig. 2a). Additionally, the absence of a visualized hypoechoic ring representing the anal sphincter (Fig. 2b) indicates the presence of anorectal atresia. US was repeated at 27 weeks of gestation. In addition to the previous ultrasound signs, the ultrasound revealed a septated median tubular cyst extended to the perineum (Fig. 3a, b), located behind the bladder (Fig. 3c). Moreover, fetal ascites (Fig. 3d) and clitoral hypertrophy (Fig. 3e) were observed. The amniotic fluid volume was normal. During the follow-up ultrasounds at 31 + weeks and 33 + weeks, the fetal biometry and growth were appropriate for her gestational age. However, the pelvic cystic mass had enlarged (Fig. 4a, b), with the top of the mass being separated from the left and right sides symmetrically (Fig. 4c,d), and the hydronephrosis had become more severe (Fig. 4e, f). At a gestation of 33 weeks + 6 days, a cesarean section was carried out due to concerns regarding the well-being of the fetus. An infant weighing 2200 g was born, exhibiting a cyanotic skin tone, limp limbs, weak breathing, and poor reflexes. Apgar scores were 4 (1 min)/6 (10 min). The baby had female genitalia. The newborn had an absent anal opening, leading to stool passing through the vestibule (Fig. 5a). Abdominal US and magnetic resonance imaging (MRI) demonstrated the presence of uterus didelphis, a vaginal septum, a direct connection between the urethra and vagina, and proximal rectal atresia. Transperineal ultrasound shows a fistula-like structure connected to the vagina and bladder, approximately 3 cm long (Fig. 5b). After inserting a cystoscope into the perineal opening, the urethral opening was found on the midventral side, while the rectal opening was located on the middorsal side. The vagina was visible on both the left and right sides, and the vaginal dome opening was observed on the posterolateral side. Cystoscopy measurements displayed that the common cavity of the urethra, vagina, and rectum was about 3 cm. The diagnosis of partial URSMS was confirmed.

On one day of birth, intestinal adhesiolysis and transverse colostomy were performed. Subsequently, laparoscopic anoplasty, urethroplasty, and then stoma closure were completed. She is undergoing regular check-ups at our Departments of Pediatric Surgery and Urology. The child, who is 2.7 years old, has normal defecation and no incontinence.

## Discussion and conclusions

Various names have been used in the literature to refer to URSMS, including the cloacal dysgenesis sequence [3], persistent cloaca [4], and cloacal malformation/anomaly [5, 6]. According to the presence or absence of perineal opening, Wheeler and Weaver [1] first divided URSMS into complete and partial types. Dannull KA et al. [7] classified cloacal malformations into six entities based on the number and location of perineal openings. These include cloacal dysgenesis, cloaca variant, posterior cloaca variant, classic cloaca (also known as persistent cloaca), posterior cloaca, and urogenital sinus. In this study, the newborn was diagnosed with partial URSMS or persistent cloaca based on detecting a single shared channel at the perineal site. The incidence of this anomaly has been reported in the literature as 2.8 per 100,000 total births [8].

At present, the pathogenesis and inheritance mechanism of URSMS remains unclear. In this case, the expectant mother had not been diagnosed with conditions other than gestational diabetes mellitus. Though previous studies have reported that high blood glucose levels during early pregnancy are associated with an increased risk of various birth defects [9], case reports on URSMS complicated by gestational diabetes mellitus are scarce [10, 11]. In this respect, the association between maternal diabetes and URSMS needs further investigation.

Escobar et al. [2] speculated that URSMS might be responsible for the incomplete separation of the cloaca by the urorectal septa and/or the failure of the urorectal septa to fuse with the cloacal membrane. Since both the urinary and reproductive systems originate in the lateral mesoderm of the body segment in the early embryo, malformations of the urinary and reproductive systems often coexist.

The external genitalia of the URSMS fetus are often indistinct and difficult to distinguish, and most female fetuses are associated with double vaginal and double uterine malformations [12]. In cases of URSMS, urine outlet obstruction and subsequent reflux into the vaginal cavity can lead to a progressive vaginal enlargement (hydrocolpos), which occurs in 30–50% of patients. This enlargement eventually results in the compression of nearby structures, such as the uretero-vesical junction, ultimately causing bilateral hydronephrosis [13]. Moreover, the backflow of urine or meconium through the fallopian tube can cause fetal peritonitis and ascites. The combination of urine and meconium can lead to calcification, thus bringing about the possibility of observing intraperitoneal calcified images.

In our case, an abdominal cyst was presented at 12 weeks gestation and resolved at 15 weeks. We hypothesized that it was probably formed by intestinal dilation. The reasons behind the early-stage dilation and its subsequent appearance as echogenic bowel at 15 weeks remain uncertain. The visibility of dilated bowel contents during the first trimester could be potentially attributed to ingesting amniotic fluid [14]. Additionally, the onset of bowel peristalsis at around 15 weeks of gestation may lead to the appearance of isoechoic meconium in the fetal bowel. Echogenic bowel persisted throughout pregnancy, which may have been caused by mixing urine and meconium. At 27 weeks, we found a separate cyst at the midline of the pelvic cavity, which was later confirmed to be a vaginal effusion after delivery. The effusion increased in late pregnancy, resulting in uterine effusion. Unfortunately, due to the sonographer’s lack of understanding about cloaca malformation, this structure was misinterpreted as intestinal dilation during the prenatal ultrasound, while the correct diagnosis of the double uterus and double vagina was not made. In our case, the symptoms became increasingly apparent in the third trimester, and hydronephrosis suddenly worsened in the late trimester due to the pressure exerted by the fluid in the vagina and uterus. An ultrasound examination at the 27th week of gestation revealed abdominal fluid accumulation and turbidity, coinciding with the initiation of meconium peritonitis. The literature suggests a significant association between fetal ascites and various factors, including preterm birth, higher birth weight z-score, birth via emergency cesarean delivery, and low Apgar scores at 1 and 5 min [15]. Our case was consistent with literature reports, which also presented with preterm labor and respiratory distress [15].

Diagnosing partial URSMS through prenatal ultrasound commonly involves identifying several characteristic findings, including an abdominal/pelvic cystic mass, hydronephrosis, oligohydramnios, distended bowel/bowel obstruction, and ascites. The diagnosis is typically considered when two or three adjacent cysts are visualized in the female fetal pelvis, corresponding to the bladder and the vagina, with the latter often exhibiting septation [16, 17]. Almost all of these ultrasound findings were observed in our case. Furthermore, we have demonstrated the sonographic evolution of abnormalities throughout gestation, particularly in early pregnancy ultrasound findings. Previous literature has reported only a few cases where fetal abdominal cysts during the initial trimester were related to anorectal malformations [18–21]. Moreover, only two cases were confirmed as cloacal malformations through autopsy [22].

URSMS represents the most severe type of anorectal malformation; therefore, early and accurate diagnosis is crucial for providing subsequent prenatal counseling and ensuring families are well-prepared for the perinatal journey. If a fetal abdominal cyst is detected between 11 and 14 weeks of gestation, the possibility of anorectal malformation should be considered. Serial ultrasound assessments provide significant advantages in monitoring the evolving abnormalities during gestation, enabling the gradual recognition of characteristics associated with the cloacal anomaly during fetal development.

## Data Availability

The datasets used and/or analyzed during the current study are available from the corresponding author upon reasonable request.

## List of abbreviations

URSMS: Urorectal septum malformation sequence
US: Ultrasonography
MRI: magnetic resonance imaging
CY: Cyst
B: Bladder
V: Vaginal
RK: Right-sided kidney
LK: Left-sided kidney
LUT: Left-sided bicornate uterus
LS: Left hydrosalpinx
R-UT: Right-sided bicornate uterus
